# Bronchoesophageal Fistula Stenting Using High-Frequency Jet Ventilation and Underwater Seal Gastrostomy Tube Drainage

**DOI:** 10.1155/2016/8175127

**Published:** 2016-09-08

**Authors:** Nitish Fokeerah, Xinwei Liu, Yonggang Hao, Lihua Peng

**Affiliations:** Department of Anesthesiology, The First Affiliated Hospital of Chongqing Medical University, No. 1, Youyi Road, Yuanjiagang, Yuzhong District, Chongqing 400016, China

## Abstract

Managing a patient scheduled for bronchoesophageal fistula repair is challenging for the anesthetist. If appropriate ventilation strategy is not employed, serious complications such as hypoxemia, gastric distension, and pulmonary aspiration can occur. We present the case of a 62-year-old man with a bronchoesophageal fistula in the left main stem bronchus requiring the insertion of a Y-shaped tracheobronchial stent through a rigid bronchoscope, under general anesthesia. We successfully managed this intervention and herein report this case to demonstrate the effectiveness of underwater seal gastrostomy tube drainage used in conjunction with high-frequency jet ventilation during bronchoesophageal fistula stenting.

## 1. Introduction

Bronchoesophageal fistula (BEF) is a pathological communication between the bronchial tree and the esophagus. BEF usually result from aerodigestive malignancies, blunt or penetrating chest trauma, chronic granulomatous infections, or ingestion of foreign bodies and corrosive substances, or it can be congenital [1, 2]. BEF is a life-threatening condition which if left untreated will ultimately lead to overwhelming sepsis and death [1]. Therefore, timely diagnosis and treatment are of paramount importance. Isolating the bronchus from the esophagus remains the basis of management of this pathology. This can be achieved through the insertion of a tracheobronchial stent (TBS). However, this intervention has various complex anesthetic implications. Nevertheless, we successfully managed the stenting of an acquired BEF using high-frequency jet ventilation (HFJV) and gastric drainage system, whereby the patient's gastrostomy tube was connected to an underwater seal bottle.

## 2. Case Report

**Figure 1:**
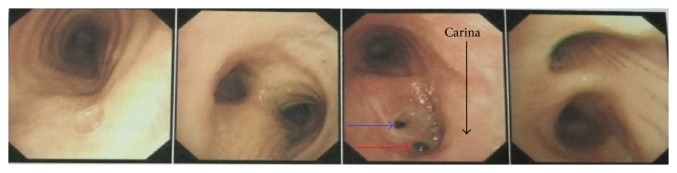
Bronchoscopy showing the presence of a bronchoesophageal fistula (blue arrow) and an ulcer (red arrow) in the left main bronchus.

**Figure 2:**
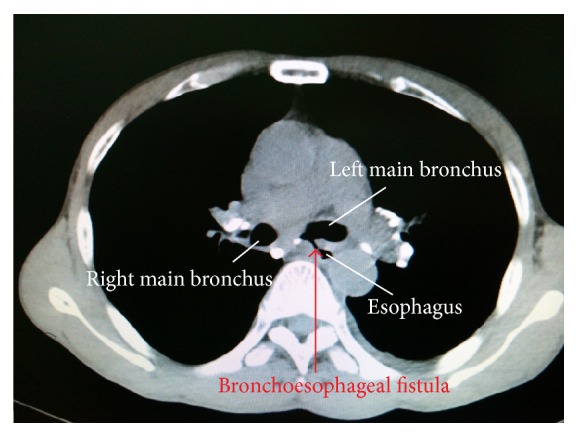
Computed tomography of the chest depicting the presence of a bronchoesophageal fistula between the left main bronchus and the esophagus.

A 62-year-old man (weight 60 kg, height 170 cm, and BMI: 20.8 kg/m^2^), chronic smoker and working as a stone breaker for the past 40 years, complained of cough and hemoptysis since 15 years and progressive shortness of breath for last one year. His symptoms got exacerbated and he was admitted to our hospital. Both fiberoptic bronchoscopy (Figure 1) and computed tomography scan of the chest (Figure 2) showed the presence of a 7 mm by 8 mm BEF in the left main stem bronchus, 3.2 cm distal to the carina. The etiology was chronic tracheobronchitis secondary to chronic nontuberculous mycobacterium (NTM) infection caused by* Mycobacterium fortuitum*. Besides the BEF, the patient also had underlying pneumonia, pneumoconiosis, and mild chronic obstructive pulmonary disease.

The patient was scheduled for the insertion of a Y-shaped covered self-expandable metal stent (CSEMS) (Micro-Tech Y-stent, Nanjing, China) through a rigid bronchoscope, under general anesthesia. Three weeks prior to the scheduled surgery, oral feeding was discontinued for both solids and liquids and a percutaneous endoscopic gastrojejunostomy was performed for enteral feeding. The patient also received total parenteral nutrition, intravenous fluids, antibiotics, and oxygen by face mask. Moreover, a thorough discussion between the interventionist and anesthetist was undertaken regarding the surgical procedure and its anesthetic implications.

**Figure 3:**
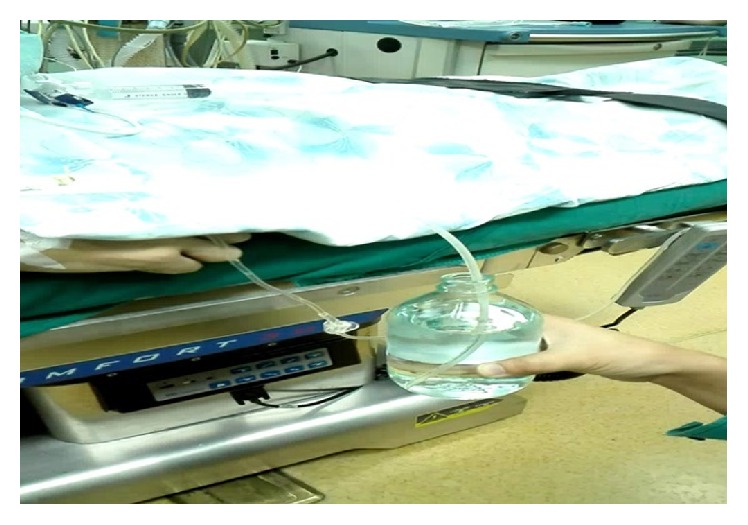
Patient lying on the operating table with his gastrostomy tube connected to an underwater seal bottle.

In the operation room, Ringer's lactate drip was started. Standard monitoring included pulse oximetry, electrocardiography, temperature, and invasive blood pressure monitoring via a left radial artery catheter. The gastrostomy tube was suctioned and connected to an underwater seal bottle containing a 10 cm column of water (Figure 3). Topical anesthesia was achieved by atomization of the nasopharynx and oropharynx with 4 mL of lidocaine 2%. The patient was preoxygenated with 100% oxygen at 5 L/min via a tight-fitting face mask for 3 minutes. Thereafter, injection penehyclidine 0.5 mg, hydrocortisone 100 mg, and omeprazole 40 mg were administered, followed by induction with propofol 100 mg and sufentanil 10 mcg. The patient was gently ventilated manually to ascertain the ability to ventilate before giving the muscle relaxant. Simultaneously, we could observe few gas bubbles in the underwater seal bottle, but ventilation was not substantially compromised, thus indicating the efficacy of the gastric drainage system. Succinylcholine 40 mg was injected and eventually the trachea was intubated with the rigid bronchoscope. Additional lidocaine was applied to the tracheobronchial tree in a “spray-as-you-go” manner via the bronchoscope. Then, the HFJV machine (KR-IV [A], Nanchang GENE Medical Devices, Jiangxi, China) was connected to the ventilating port of the bronchoscope. Ventilation parameters included frequency 100/min, driving pressure 25 psi, I : E ratio 1 : 2, and FiO_2 _0.7. Anesthesia was maintained with an infusion of propofol 40–60 mcg/kg/min and remifentanil 0.15–0.2 mcg/kg/min. The case proceeded uneventfully, with serial blood gas analysis performed (Table 1) to assess ventilation. No hypoxemia took place and patient remained hemodynamically stable throughout the procedure. However, transitory hypercapnia occurred (Table 1). This case was managed without the use of long acting muscle relaxant, as the patient did not have airway reflexes such as coughing and laryngospasm, patient remained apneic, and the condition for bronchoscopy was good during the intervention.

**Figure 4:**
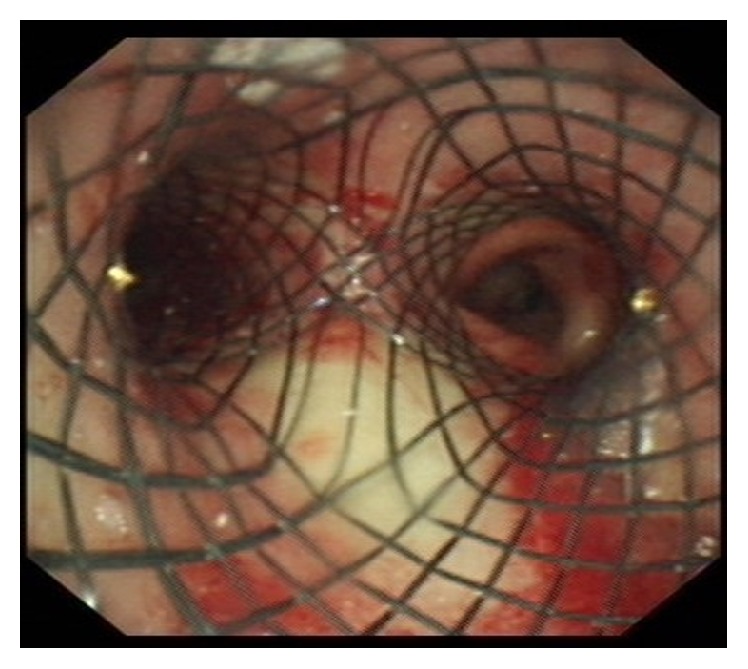
Correct position of the Y-shaped tracheobronchial stent, sealing the left bronchoesophageal fistula.

Once the stent (size: 18 × 30 mm – L 14 × 30 mm – R 14 × 10 mm) was deployed and its correct position confirmed (Figure 4), the bronchoscope was removed and HFJV stopped. An “i-gel” laryngeal mask airway (LMA) was inserted and conventional ventilation started. The patient was shifted to postanesthesia care unit, where emergence from anesthesia and recovery were quick and uneventful. On postoperative day 2, bronchoscopy showed correct position of the stent and patient was allowed to start oral feeding. Four days after the intervention, the patient was clinically stable and discharged from the hospital. One month later, the patient attended the outpatient department; he was asymptomatic and bronchoscopy showed that the CSEMS was in the correct position, sealing the BEF. The whole procedure including anesthesia induction and emergence took 55 minutes to be accomplished. Surgical time was 30 minutes.

## 3. Discussion

Anesthesia for patients with BEF requiring tracheobronchial stent insertion is challenging for the anesthetist. The challenges include a shared airway, underlying pulmonary pathologies, and coexisting advanced enterorespiratory malignancies. Furthermore, the use of intermittent positive pressure ventilation (IPPV) causes leakage of air through the BEF leading to two main complications. Firstly, distention of the stomach and probable gastric regurgitation leading to pulmonary soiling. Secondly, ventilation of the fistula accounts for inadequate lung ventilation, hypoxemia, atelectasis, and carbon dioxide (CO_2_) retention. This case report highlights the various anesthetic considerations for the successful management of BEF.

HFJV has been very effective in this case. In contrast to conventional ventilation, HFJV delivers very small tidal volume (*V*_*T*_) at high frequency, at a lower airway pressure [3, 4], thus decreasing gas outflow through the fistula. Typically, *V*_*T*_ delivered by HFJV is less than the anatomical dead space. Moreover, there is minimal diaphragmatic excursion [3], which provides a motionless surgical field. However, HFJV may cause inadequate CO_2_ elimination and hypercapnia. In this case, the maximum PaCO_2_ level was 82 mmHg, duration of hypercapnia was about 15 minutes, and no hypoxemia took place. Hence, this permissive hypercapnia did not require temporary interruption of the HFJV to provide controlled ventilation. Cheng et al. [5] reported that transient hypercapnia (PaCO_2_ < 100 mmHg) does not prolong the recovery time, nor is it associated with serious complications. Another effective method to assess ventilation during interventional bronchoscopy is transcutaneous capnography (Ptc_CO2_). In addition, Gupta et al. [6] reported that using heliox (mixture of 80% helium and 20% oxygen) improves CO_2_ elimination during HFJV.

Furthermore, we used an underwater seal drainage system, whereby the patient's gastrostomy tube was connected to an underwater seal bottle containing a 10 cm column of water. This strategy increases the resistance to gas flow from the airway to the esophagus. Therefore, gas will leak through the fistula only if the airway pressure exceeds the underwater seal pressure (10 cm H_2_O). There are several benefits of this technique. Firstly, it accounts for better pulmonary ventilation by minimizing gas outflow through the fistula. Secondly, any gas leak to the stomach will be drained through the gastrostomy tube, not increasing the hydrostatic pressure of the stomach, hence no gastric regurgitation. To the best of our knowledge, a gastric underwater seal has not been used in the management of BEF in adults. This technique has been used in the management of congenital tracheoesophageal fistula (TEF) in infants. Donn et al. [7] successfully ventilated two newborns with congenital TEF, using HFJV in conjunction with gastric underwater seal. Likewise, Sosis and Amoroso [8] effectively ventilated an infant with congenital TEF using positive pressure ventilation, with the newborn's gastrostomy tube connected to an underwater seal. This methodology has also been demonstrated to alleviate gastric distension caused by aerophagia by using underwater seal nasogastric tube drainage [9].

There are alternative means of providing effective ventilation for the management of enterorespiratory fistulas. For instance, manual jet ventilation (MJV) can be used. MJV offers more efficient ventilation and CO_2_ elimination compared to HFJV, due to adequate expiratory time and better chest and lung recoil during expiration [10]. Dolan and Moore [11] ventilated the lungs by passing the jet catheter of the HFJV distal to the fistula, thus minimizing gas leakage. Other options include endobronchial intubation, Univent endobronchial blocker, and double lumen tube to the unaffected bronchus [12, 13]. More complex fistulas have been managed successfully with bilateral endobronchial intubation [14], extracorporeal membrane oxygenation [15], and cardiopulmonary bypass [16]. Some authors reported sealing the fistula prior to the intervention, using a modified esophageal balloon [17], or a Sengstaken–Blakemore tube [18]. Garg et al. [19] reported two cases in children where they sealed the TEF using Fogarty and Foley catheter. In our case, if the surgeon would not have used rigid bronchoscope, we could have used an LMA, avoid muscle relaxant, maintain the patient's spontaneous breathing, and under FOB guidance place the jet catheter of a MJV into the right bronchus to provide intermittent ventilation if required. The jet catheter must be withdrawn upwards into the trachea at the time of stent deployment.

The anesthetic goal for stenting a BEF is to use drugs which are quick in onset, short acting, and readily eliminated. Muscle relaxant is needed to provide optimal relaxation for insertion of the rigid bronchoscope and manipulation of the airway, in order to prevent coughing, laryngospasm, and chest rigidity. We used succinylcholine and performed a rapid sequence induction (RSI). However, succinylcholine is short acting and eventually a top-up dose of another muscle relaxant may be necessary if the patient shows signs of discomfort, starts spontaneous respiration which is hindering with the surgery, or develops airway reflexes. Ideally, rocuronium is the muscle relaxant of choice for this type of intervention. We may use 1.2 mg/kg of rocuronium and perform a RSI. At the end of the surgery, we can promptly antagonize the rocuronium with sugammadex. However, sugammadex is not available in our hospital. Regarding anesthesia maintenance, Wang et al. [20] described that propofol and remifentanil TCI in association with HFJV for bronchoscopy procedures provide multiple benefits. Total intravenous anesthesia (TIVA) is ideal, since the airway remains essentially open to the atmosphere and use of inhaled anesthetics will lead to pollution of the operation room. Furthermore, in the authors' case, atomization of the airway with lidocaine suppressed the cough reflex, prevented laryngospasm, and reduced the need for systemic opioids and sedatives, hence favoring early recovery.

After the placement of the stent, we should advocate the early return of spontaneous respiration, early extubation, and weaning from the ventilator. Therefore, after the intervention, insertion of an LMA is preferred to an endotracheal tube. Endotracheal intubation requires a deeper depth of anesthesia, which will prolong the recovery, and it may also cause coughing and laryngospasm which may cause displacement of the newly placed stent.

Besides all these aspects, a key determining factor is constant and effective communication and cooperation between the interventionist and anesthetist, which plays a pivotal role in the success of this surgery.

In conclusion, anesthetic management for BEF stenting is challenging. However, we successfully managed the surgery using HFJV in conjunction with an underwater seal gastrostomy tube drainage system. The latter has played a crucial role in the success of our case and is a novel strategy for BEF management in adults. Nevertheless, we will consider enhancing our management, in view of optimizing our anesthetic plan for similar eventual interventions. Various other ways to manage a case of BEF have been reported in medical literature. Anyhow, whatever method is chosen, a planned and multidisciplinary approach to successfully tackle this complex issue of bronchoesophageal fistula will be required.

## Figures and Tables

**Table 1:** Serial arterial blood gas analyses.

	Enter OR	Preinduction	15 minutes after HFJV	Stent inserted	HFJV stopped	Entered PACU	Left PACU
PaO_2_ (mmHg)	75	278	244	105	149	387	110
PaCO_2_ (mmHg)	38	38	62	76	82	51	42
pH	7.40	7.48	7.32	7.25	7.20	7.34	7.37
SpO_2_ (%)	97	100	99	97	99	100	99

OR, operation room; HFJV, high frequency jet ventilation; PACU, postanesthesia care unit; PaO_2_, partial pressure of oxygen in blood; PaCO_2_, partial pressure of carbon dioxide in blood; SpO_2_, peripheral capillary oxygen saturation.
